# The foreign socratic intensive care medicine curricula

**DOI:** 10.1186/2197-425X-3-S1-A866

**Published:** 2015-10-01

**Authors:** SJ Shepherd, K Donadello, S Thiessen, N Aissaoui, B Bollen Pinto, G De Pascale, M Hilty, K Lane, M Mendoza, P Schellongowski, F Weidanz, B Weiss, J Werner, A Wong, L Prisco

**Affiliations:** NEXT Committee, European Society of Intensive Care Medicine, Brussels, Belgium

## Introduction

European Intensive Care Medicine (ICM) trainees and young specialists daily face the distinct local differences that exist in the minimum knowledge, skills, duration of training and non-technical behaviours which define a specialist. While ICM can be recognised as a 'particular competence' for those who have completed the appropriate training in most European countries, consistent heterogeneity may affect the potential to perform ICM research and to move across Europe.

## Objectives

To characterise training patterns across Europe and the perception of young intensivists on research, mentoring and mobility.

## Methods

A web-based multi-question survey (SurveyMonkey^®^) was prepared and delivered via e-mail to all ESICM members, so as to be received by all related ICM trainees and young specialists (within five years from graduation). Descriptive questions and 5-point Likert scale ones were used. The survey was run for one year, thereafter the collected data were anonymously analyzed (Microsoft Excel 2013). Data are presented as percentage and mean ± SD.

## Results

Among 392 respondents, 88% were interested in research, but 33% of them could not perform it, mostly because of lack of funding/support or work overload. 55% were actually doing research, mainly clinical but not linked to a specific university degree, with less than 5 dedicated hours per week and for less than 1 consecutive year. 93% of researches did not get any additional funding. 207 persons had peer-review original publications, 50% ≤2, 75% ≤4. Among 308 respondents, 83% did not hold a university teaching position, 45% envisioned an academic career and 35% believed that working abroad would be essential for it. Formal mentoring may be lacking also in academic settings: 26% of young intensivists motivated towards pursuing a research and/or academic career found inadequate support in terms of training in methodology, supervision or funding. 89% of young intensivists foresaw a period of training abroad yet in only around two-thirds of areas were local training programmes able to offer this opportunity. The main barriers were recognized in burocracy, family commitments and finalcial support.

## Conclusions

ICM trainees and young specialists are research, mentoring and mobility demanding. Many regions operate an apprenticeship model, yet formal mentoring relationships was identified in only than one third of European trainees' careers. Moving may be a necessary requirement in order to develop skills otherwise not reachable at home institution. International mutual recognition would facilitate intensivist mobility and improve multidisciplinary ICM core curricula.

## Grant Acknowledgment

This survey was endorsed by ESICM and did not receive any grant or funding.

